# A chromosome-level reference genome assembly and a full-length transcriptome assembly of the giant freshwater prawn (*Macrobrachium rosenbergii*)

**DOI:** 10.1093/g3journal/jkae146

**Published:** 2024-07-08

**Authors:** Wirulda Pootakham, Kanchana Sittikankaew, Chutima Sonthirod, Chaiwat Naktang, Tanaporn Uengwetwanit, Wasitthee Kongkachana, Kongphop Ampolsak, Nitsara Karoonuthaisiri

**Affiliations:** National Center for Genetic Engineering and Biotechnology (BIOTEC), National Science and Technology Development Agency (NSTDA), Pathum Thani 12120, Thailand; National Center for Genetic Engineering and Biotechnology (BIOTEC), National Science and Technology Development Agency (NSTDA), Pathum Thani 12120, Thailand; National Center for Genetic Engineering and Biotechnology (BIOTEC), National Science and Technology Development Agency (NSTDA), Pathum Thani 12120, Thailand; National Center for Genetic Engineering and Biotechnology (BIOTEC), National Science and Technology Development Agency (NSTDA), Pathum Thani 12120, Thailand; National Center for Genetic Engineering and Biotechnology (BIOTEC), National Science and Technology Development Agency (NSTDA), Pathum Thani 12120, Thailand; National Center for Genetic Engineering and Biotechnology (BIOTEC), National Science and Technology Development Agency (NSTDA), Pathum Thani 12120, Thailand; Aquatic Animal Genetics Research and Development Division, Department of Fisheries, Ministry of Agriculture and Cooperatives, Pathum Thani Aquatic Animal Genetics Research and Development Center, Pathum Thani 12120, Thailand; National Center for Genetic Engineering and Biotechnology (BIOTEC), National Science and Technology Development Agency (NSTDA), Pathum Thani 12120, Thailand; International Joint Research Center on Food Security, 113 Thailand Science Park, Phahonyothin Road, Khlong Nueng, Khlong Luang, Pathum Thani 12120, Thailand; Institute for Global Food Security, Queen’s University, Belfast, Biological Sciences Building, 19 Chlorine Gardens, Belfast BT9 5DL, UK

**Keywords:** *Macrobrachium rosenbergii*, chromosome-scale genome assembly, Hi-C, transcriptome, Iso-seq, alternative splicing

## Abstract

The giant freshwater prawn (*Macrobrachium rosenbergii*) is a key species in the aquaculture industry in several Asian, African, and South American countries. Despite a considerable growth in its production worldwide, the genetic complexities of *M. rosenbergii* various morphotypes pose challenges in cultivation. This study reports the first chromosome-scale reference genome and a high-quality full-length transcriptome assembly for *M. rosenbergii.* We employed the PacBio High Fidelity (HiFi) sequencing to obtain an initial draft assembly and further scaffolded it with the chromatin contact mapping (Hi-C) technique to achieve a final assembly of 3.73-Gb with an N50 scaffold length of 33.6 Mb. Repetitive elements constituted nearly 60% of the genome assembly, with simple sequence repeats and retrotransposons being the most abundant. The availability of both the chromosome-scale assembly and the full-length transcriptome assembly enabled us to thoroughly probe alternative splicing events in *M. rosenbergii*. Among the 2,041 events investigated, exon skipping represented the most prevalent class, followed by intron retention. Interestingly, specific isoforms were observed across multiple tissues. Additionally, within a single tissue type, transcripts could undergo alternative splicing, yielding multiple isoforms. We believe that the availability of a chromosome-level reference genome for *M. rosenbergii*, along with its full-length transcriptome, will be instrumental in advancing our understanding of the giant freshwater prawn biology and enhancing its molecular breeding programs, paving the way for the development of *M. rosenbergii* with valuable traits in commercial aquaculture.

## Introduction

The giant freshwater prawn (*Macrobrachium rosenbergii*), the largest prawn within the *Macrobrachium* genus, holds significant economic importance as an aquaculture species in several Asian, African, and South American countries (Yang *et al*. 2012; Cao *et al*. 2017). *Macrobrachium rosenbergii* serves as a healthy dietary source for protein, lipids, and carbohydrates (Marques *et al*. 2016; Wu *et al*. 2021). Despite a substantial global production increase over the past 2 decades, reaching 273,738 tons in 2019 (FAOSTAT website; Jiang *et al*. 2020), its cultivation faces challenges mainly arising from the existence of various morphotypes and their social interactions. In general, male individuals exhibit faster growth compared with their female counterparts (Liu *et al*. 2015). However, mature males of different morphotypes such as small male, orange claw, blue claw, and old blue claw display a wide range of size variations (Supplementary Fig. 1; Tang *et al*. 2020; Ibrahim *et al*. 2023). Previous attempts to unravel the genetic regulations of these different morphotypes involved employing short-read RNA sequencing (RNA-seq) techniques. These studies aimed to investigate the mechanisms underlying growth differences among male morphotypes (Tang *et al*. 2020), identify candidate genes involved in sex development (Ren et al. 2021), and study the effects of androgenic gland ablation on gonad development (Ying *et al*. 2022) and of transcriptome changes on immune response exposure and stress (Ding *et al*. 2018; Gao *et al*. 2020; Ou *et al*. 2021). Unfortunately, the full exploitation of gene expression data was hindered by the absence of a high-quality reference genome assembly.

In this study, we present the first chromosome-scale reference genome assembly along with the high-quality full-length transcriptome assembly in *M. rosenbergii.* We employed the HiFi long-read PacBio sequencing to generate the preliminary assembly, which we further scaffolded to the chromosome level using the chromatin contact mapping (Hi-C) approach. The transcriptome assembly, derived from 13 organs and hemocytes (HCs), utilized long-read Pacific Biosciences (PacBio) isoform sequencing (Iso-seq). The PacBio Iso-seq platform captures full-length transcripts from their 5′ ends to poly(A) tails in single long reads. This approach has proven successful in obtaining reference transcriptome assemblies for various organisms lacking reference genome sequences (Pootakham *et al*. 2020; Carneiro *et al*. 2021; Guo *et al*. 2022). We believe that this high-quality reference genome and transcriptome assemblies serve as valuable resources for genomic studies and breeding programs for *M. rosenbergii*.

## Materials and methods

### Sample collection

*Macrobrachium rosenbergii* shrimps were cultured at the Aquaculture Genetics Research and Development Center, Pathum Thani, Thailand. For whole-genome sequencing, muscles were collected from a 5-month-old male shrimp and immediately frozen in liquid nitrogen and stored at −80°C until use. For transcriptome sequencing, a total of 12 organs and HCs were collected from three 5-month-old male shrimps and ovaries from three 4-month-old female shrimps. The following organs were dissected and immediately frozen in liquid nitrogen: gill (GI), heart (HE), testis (TT), ovary (OV), androgenic gland (AG), hepatopancreas (HP), stomach (ST), intestine (IN), thoracic ganglion (TG), abdominal ganglion (ABG), eyestalk (ES), pleopods (Pl), and muscle (MU). Hemolymph was centrifuged at 3,000 rpm at 4°C for 5 min to pellet the HCs. All samples were stored at −80°C until RNA extraction. This study was performed in accordance with the recommendations of Animal Research Ethics Guidelines, and the protocol was approved by the National Center of Genetic Engineering and Biotechnology Animal Research Ethics Committee (approval number BT-IACUC-RF01-10-01).

### DNA and RNA extraction

Frozen muscle tissue was ground in liquid nitrogen, and genomic DNA was extracted using a Genomic Tip 100/G kit (Qiagen, USA), as previously described (Angthong *et al*. 2020). DNA quantity was measured using a NanoDrop ND-8000 spectrophotometer and a Qubit dsDNA BR Assay kit (Invitrogen, USA) using a Qubit fluorometer. The DNA quality and integrity were visualized under pulsed field gel electrophoresis at 80 V for 9 h in 0.5× KBB buffer (51 mM Tris, 28 mM TASP, 0.08 mM EDTA, pH 8.7; Sage Science, USA) containing SYBR Safe DNA gel staining (Invitrogen). Total RNA was extracted using the TRI-reagent according to the manufacturer's instruction (Molecular Research Center, USA) and DNase treated with RNase-free DNase (0.5 U/µg total RNA at 37°C for 30 min; Promega, USA). The treated RNA was quantified using a Qubit fluorometer (Invitrogen), and RNA quality was assessed by agarose gel electrophoresis and using Agilent 2100 Bioanalyzer (Supplementary Table 1; Agilent, Santa Clara, CA, USA).

### Genome and transcriptome sequencing

For the reference genome sequencing, the PacBio SMRTbell library (∼20 kb) for PacBio Sequel was constructed using SMRTbell Express Template Prep Kit 2.0 (PacBio, Menlo Park, CA, USA) and following the manufacturer-recommended protocol. The library was bound to polymerase using Sequel II Binding Kit 2.0 (PacBio) and loaded onto PacBio Sequel II. Sequencing was performed on PacBio Sequel II 8M SMRT cells. For transcriptome sequencing, 1 µg of DNase-treated RNA sample from each tissue was used for cDNA synthesis with a SMARTer PCR cDNA Synthesis kit (Clontech, USA). Iso-seq library construction was carried out using a SMRTbell Template Prep Kit 1.0-SPv3 protocol (Pacific Biosciences, Menlo Park, CA, USA). Samples from each tissue were barcoded, pooled into a single library, and sequenced on the PacBio Sequel II platform. The PacBio sequencing was outsourced to Omics Drive, Singapore.

### Hi-C (Dovetail Omni-C) library preparation and sequencing

A chromosome conformation capturing technique (Hi-C) was conducted by Dovetail Genomics (Scott Valley, USA) to scaffold *M. rosenbergii* preliminary assembly into a chromosome-level assembly. A DNase Hi-C library was prepared, as previously described in Ma *et al*. (2018). Briefly, chromatin was fixed in place with formaldehyde in the nucleus and then extracted. The fixed chromatin was digested with DNAseI, and chromatin ends were repaired and ligated to a biotinylated bridge adapter, followed by proximity ligation of adapter-containing ends. After proximity ligation, cross-links were reversed and the DNA purified from protein. The purified DNA was treated to remove biotin that was not internal to the ligated fragments. Sequencing libraries were generated using NEBNext Ultra enzymes and Illumina-compatible adapters. Biotin-containing fragments were isolated using streptavidin beads before the process of PCR enrichment of each library. The libraries were sequenced on an Illumina HiSeq X to produce 116,073,039 read pairs.

### De novo assembly and Hi-C (Omni-C) scaffolding of the *M. rosenbergii* genome

A total of 1,228 Gb PacBio raw reads (301.1× coverage) were subjected to trimming and read correction, resulting in 58.39 Gb high-accuracy circular consensus sequencing (CCS) data (14.3×). The PacBio CCS reads were used as an input to Hifiasm v0.15.4-r347 with default parameters (Cheng *et al*. 2021). The blast results of the Hifiasm output assembly against the nt database were used as input for blobtools v1.1.1 (Laetsch and Blaxter 2017), and scaffolds identified as possible contamination were removed from the assembly. We applied an error correction tool racon (https://github.com/isovic/racon) to improve the quality of the reads in low-coverage regions. Finally, purge_dups v1.2.5 (Guan *et al*. 2020) was used to remove haplotigs and contig overlaps. A PacBio draft assembly was used as an input for the subsequent scaffolding with HiRise, a software pipeline designed specifically for using proximity ligation data to scaffold genome assemblies (Putnam *et al*. 2016).

The input de novo assembly and Dovetail Omni-C library reads (136.7 Gb, 33.5×) were used as input data for HiRise, a software pipeline designed specifically for using proximity ligation data to scaffold genome assemblies (Putnam *et al*. 2016). Dovetail Omni-C library sequences were aligned to the draft input assembly using bwa (Li and Durbin 2010). The separations of Dovetail Omni-C read pairs mapped within draft scaffolds were analyzed by HiRise to produce a likelihood model for genomic distance between read pairs, and the model was used to identify and break putative misjoins, to score prospective joins, and to make joins above a threshold.

### Genome assembly evaluation

The quality of the final genome assembly was evaluated by aligning transcriptome (Iso-seq) data from 14 tissues (see the Sample collection section) using minimap version 2.17 (Li 2018). In addition, the presence and the completeness of the orthologs were determined using the Benchmarking Universal Single-Copy Orthologues (BUSCO) version 5.4.4 (Simão *et al*. 2015) and the Arthropoda OrthoDB release 10 (Kriventseva *et al*. 2015).

### Identification of repetitive sequences and gene annotation

The following pipeline was employed to identify repetitive sequences in *M. rosenbergii*, *Macrobrachium nipponense*, *Penaeus monodon*, *Hyalella azteca*, *Litopenaeus vannamei*, and *Parhyale hawaiensis*. Repbase library (Bao *et al*. 2015) was employed to identify repeats de novo from the genome assembly using RepeatModeler (Flynn *et al*. 2020). RepeatMasker (Tempel 2012) was subsequently used to identify repeats in the genome assembly and mask them by lowercase letters (soft masking).

To annotate the protein-coding sequences within our research, we utilized the EVidenceModeler (EVM) software, version 1.1.1, as described by Haas *et al*. (2008). This tool allows for the integration of different types of evidence into a cohesive annotation system. Our approach combined homology-based, RNA-based, and ab initio prediction methods for annotating the genome assembly. The IsoSeq3 pipeline (https://github.com/ylipacbio/IsoSeq3) was used to process SMRT-sequencing (Iso-seq) data from 14 different tissues. Briefly, the IsoSeq3 pipeline removed the primer sequences, trimmed the adapter/barcode sequences, and filtered out the concatemers as well as immature transcript sequences without the poly(A) tails. Polishing was carried out using the tool *polish* in the Isoseq3 pipeline, and only high-quality reads supported by at least 2 full-length nonchimeric reads with a predicted sequence accuracy of >99% were used in the downstream analyses. For the transcript-based predictions, Iso-seq reads were grouped into clusters at 97% similarity with CD-HIT (version 4.8.1; Fu *et al*. 2012). The longest open-reading frame (ORF) from each cluster was selected for further analysis. These selected ORFs were then aligned to the genome assembly using PASA version 2.5.3 (Haas *et al*. 2008) and GMAP version 2020-09-12 (Wu and Watanabe 2005). We also utilized protein sequences from related species such as *H. azteca* (GCF_000764305.2), *L. vannamei* (GCF_003789085.1), *P. monodon* (GCF_015228065.2), and *M. nipponense* (GCA_015104395.2). These sequences were aligned to our genome assembly using the Analysis and Annotation Tool (AAT), as outlined by Huang *et al*. (1997). For the ab initio predictions, we employed the Augustus software (version 3.2.1; Stanke *et al*. 2004) trained with the data obtained from *H. azteca*, *L. vannamei*, *P. monodon*, and *M. nipponense* and incorporated the PASA transcript alignments for further refinement. In the final step, EVM integrated these 3 types of evidence—assigning weights of 5 to PASA, 1 to GMAP, 0.5 to AAT, and 0.1 to Augustus predictions—to produce the consensus gene models.

Protein functions were predicted using InterProScan (Blum *et al*. 2021). Gene ontology (GO) terms and Kyoto Encyclopedia of Genes and Genomes pathway annotation were assigned to the sequences (Moriya *et al*. 2007; Kanehisa and Sato 2020). Infernal version 1.1 (Nawrocki and Eddy 2013) was used to perform a homology search and annotate noncoding RNA-seqs.

### Identification of alternative splicing events

To explore alternative splicing events in *M. rosenbergii*, we employed the TAPIS pipeline (version 1.2.1; Abdel-Ghany *et al*. 2016) and SpliceGrapher program (Rogers *et al*. 2012) to identify the following events: alternative 5′ donor-site selection, alternative 3′ acceptor-site selection, exon skipping, and intron retention.

### Phylogenetic analyses and comparative genomics

OrthoFinder version 2.4.0 (Emms and Kelly 2019) was employed to extract 210 single-copy orthologous proteins. Subsequently, gene alignments were performed using MUSCLE version 3.8 (Edgar 2004). The best-fit models of amino acid substitution were estimated, and a maximum-likelihood phylogenetic tree was reconstructed using Mega version 10.1.8 (Tamura *et al*. 2011). The tree's robustness was assessed through 1,000 replicates of bootstrap analysis utilizing the WAG + G + F substitution model based on Mega’s outcomes.

## Results

### Genome assembly of *M. rosenbergii*

**Fig. 1.**
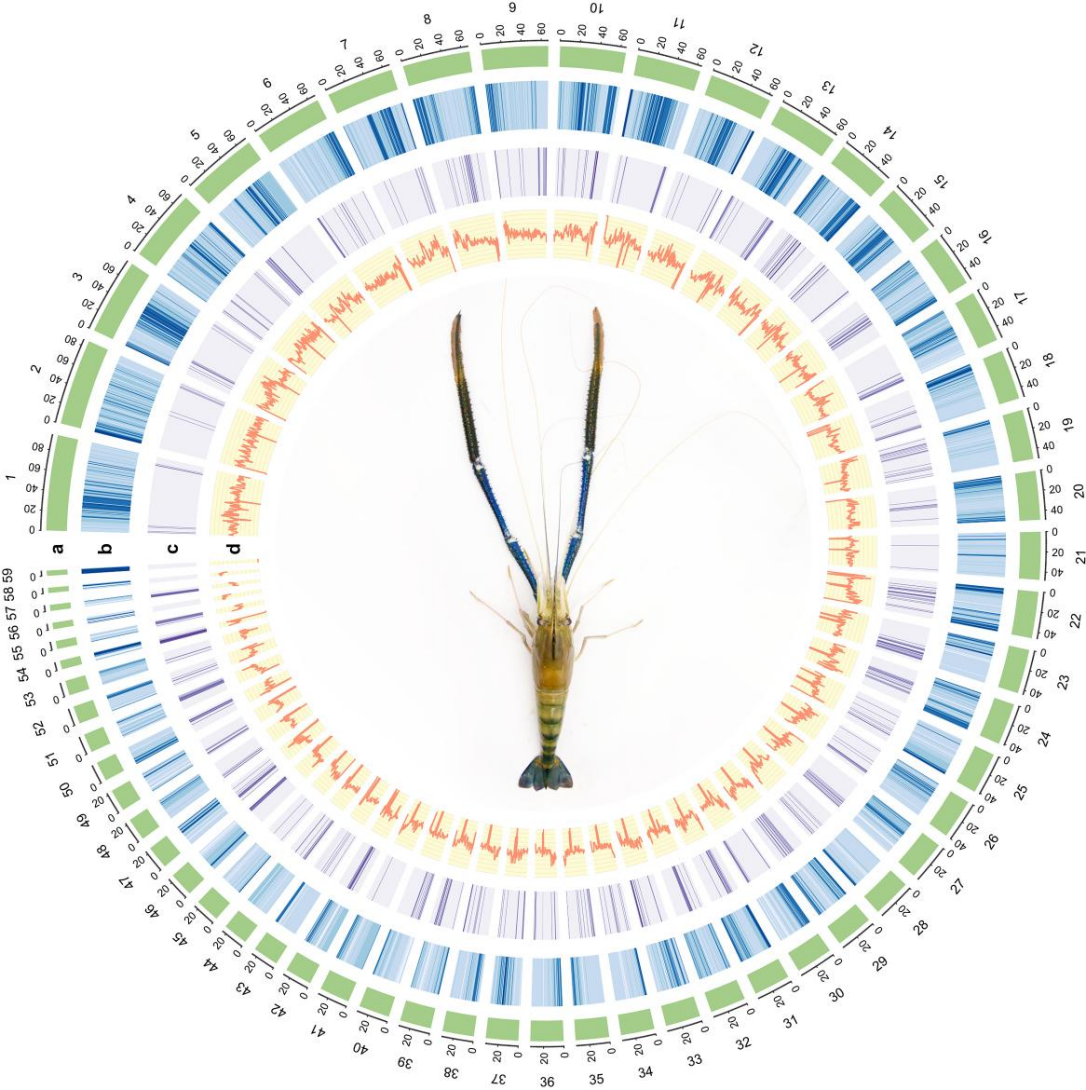
A genomic landscape of *M. rosenbergii.* a) A physical map of 59 pseudomolecules (chromosomes) numbered according to size (in Mb). b) Repeat content represented by the proportion of genomic regions covered by repetitive sequences in 250-kb windows. c) Gene density represented by the number of genes in 250-kb windows. d) GC content represented by the percentage of G + C bases in 250-kb windows.

To acquire a high-quality genome assembly, we employed the PacBio HiFi sequencing technology to produce 1,228 Gb (∼301-fold coverage of the estimated genome size) of raw reads (read number: 4,259,973; average N50: 13,950 kb). Genome assembly was carried out using Hifiasm, and the preliminary genome of 3.73 Gb was generated with a 249-kb contig N50. Our assembly results demonstrated that the size of the assembled giant freshwater prawn was relatively close to the estimated genome size by the flow cytometry (4.08 Gb; Levy *et al*. 2019). To further improve the contiguity of our preliminary assembly, we employed the in vivo chromosome fixation technique (Hi-C) based on the information from 116,073,039 read pairs (136.7 Gb of Hi-C data) to generate a chromosome-level assembly. The final assembly encompassed 3,739,422,449 bases with an N50 scaffold length of 33.6 Mb (Table 1) and contained 59 pseudomolecules (hereafter referred to as chromosomes numbered according to sizes), corresponding to the haploid chromosome number in *M. rosenbergii* (2*n* = 108; Fig. 1; Supplementary Fig. 2).

**Table 1.** Assembly statistics of the *M. resenbergii* genome^a^.

	PacBio	PacBio + Hi-C
N50 contigs/scaffold size (bases)	249,506	33,688,259
L50 contigs/scaffold number	3,288	35
N75 contigs/scaffold size (bases)	100,791	897,491
L75 contigs/scaffold number	9,238	290
N90 contigs/scaffold size (bases)	44,296	127,752
L90 contigs/scaffold number	17,585	2,440
Assembly size (bases)	3,738,295,162	3,739,422,449
Number of contigs/scaffolds	29,644	10,042
Number of contigs/scaffolds ≥100 kb	9,314	3,067
Number of contigs/scaffolds ≥1 Mb	337	268
Number of contigs/scaffolds ≥10 Mb	0	53
Longest contigs/scaffold (bases)	6,547,088	95,859,317
% *N*	0	2,113,600
GC content (%)	30.36	30.35
BUSCO evaluation (% completeness)	94.4%	94.8%
Complete and single copy	86.3%	88.2%
Complete and duplicated	3.1%	2.7%
Fragmented	5.1%	3.9%
Missing	5.5%	5.2%

^*a*^Sequences generated by the initial assembly of PacBio HiFi reads are in contigs, whereas those generated by Hi-C scaffolding are in scaffolds.

We performed an evaluation of our assembly quality by aligning Iso-seq reads to the assembled genome, and 96.33% of *M. rosenbergii* Iso-seq transcripts were mapped to the genome. To further assess the completeness of the gene space in each assembly, we used the BUSCO software to check the gene content using an arthropoda_odb10 database (1,013 genes; Simão *et al*. 2015). Our gene prediction recovered 90.9% of the highly conserved orthologs in the Arthropod lineage with 94.8% identified as “complete and single-copy,” 2.7% as “complete and duplicated” and 3.9% as “fragmented,” while 5.2% of the conserved orthologs were missing from the assembly (Table 1). This evidence supported the high-quality assembly of the *M. rosenbergii* genome.

### Gene annotation

We integrated 3 different approaches including an ab initio prediction, a homology-based search, and a transcript-based prediction in the annotation pipeline to predict 34,203 gene models and 29,181 protein-coding genes in *M. rosenbergii*. Gene sequences in *M. rosenbergii* were preferentially distributed near the telomeres for most of the chromosomes (Fig. 1; Supplementary Tables 2 and 3). The top 3 most prevalent GO terms associated with cellular components were nucleus, cytoplasm, and cytosol (Supplementary Fig. 3), while the largest categories of genes annotated to molecular function were protein binding, protein homodimerization activity and ATP hydrolysis activity. The most common terms for biological processes were multiorganism reproductive process, obsolete regulation of cellular macromolecule biosynthetic process, and obsolete cellular macromolecule metabolic process (Supplementary Fig. 3). In addition, noncoding RNAs were also annotated using homology searches, and there were 1,252 ribosomal RNA, 1,957 transfer RNA, 802,741 microRNA, and 12,981 small nuclear RNA (Supplementary Table 4).

### Identification of repetitive elements

**Fig. 2.**
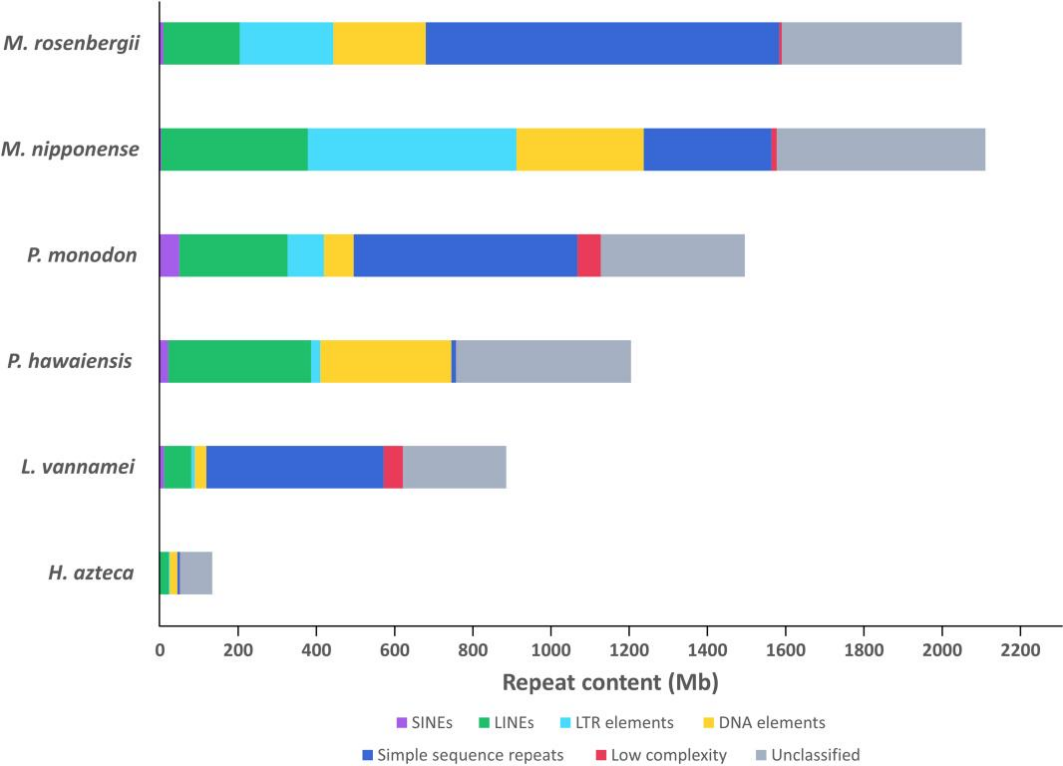
Repetitive elements in *M. rosenbergii* and other related species.

We employed a combination of a de novo repeat identification tool, RepeatModeler, and homology search tools to analyze repetitive sequences in *M. rosenbergii*, *M. nipponense*, *P. monodon*, *H. azteca*, *L. vannamei*, and *Pa. hawaiensis*. The total length of repetitive sequences accounted for 57.54% of the assembly (2.15 Gb; Table 2), which was higher than the proportion of repetitive sequences observed in *M. nipponense* (45.6%; Jin *et al*. 2021). Interestingly, simple sequence repeats represented the majority of classified repetitive elements in the *M. rosenbergii* genomes, comprising almost a quarter of the assembly (904 Mb) and 42% of the total repeat contents (Fig. 2). Likewise, a noticeably large proportion of simple sequence repeats was observed in *P. monodon* and *L. vannamei.* Retrotransposons also occupied a significant portion of the *M. rosenbergii* genome (443 Mb; 11.86%), with long terminal repeats (LTRs) being the most prevalent class (54% of all retrotransposons). The LTRs could be categorized into 2 main classes: the *Copia-* and *Gypsy-*like elements, which represented 0.24 and 10.27% of the total repeat contents, respectively (Table 2).

**Table 2.** Repeat contents in the *M. rosenbergii* genome.

Types of repeats	Bases (Mb)	% of the assembly	% of total repeats
DNA transposons	235,762,910	6.30	10.95
Retrotransposons			
LINE	194,853,557	5.21	9.05
SINE	9,407,001	0.25	0.43
LTR: *Copia*	5,258,439	0.14	0.24
LTR: *Gypsy*	220,065,862	5.89	10.27
LTR: Others	13,983,259	0.37	0.64
Simple sequence repeats	904,474,219	24.19	42.03
Others	567,837,820	15.19	26.4
Total	2,151,703,067	57.54	

### Comparative genomics and phylogenetic analyses

**Fig. 3.**
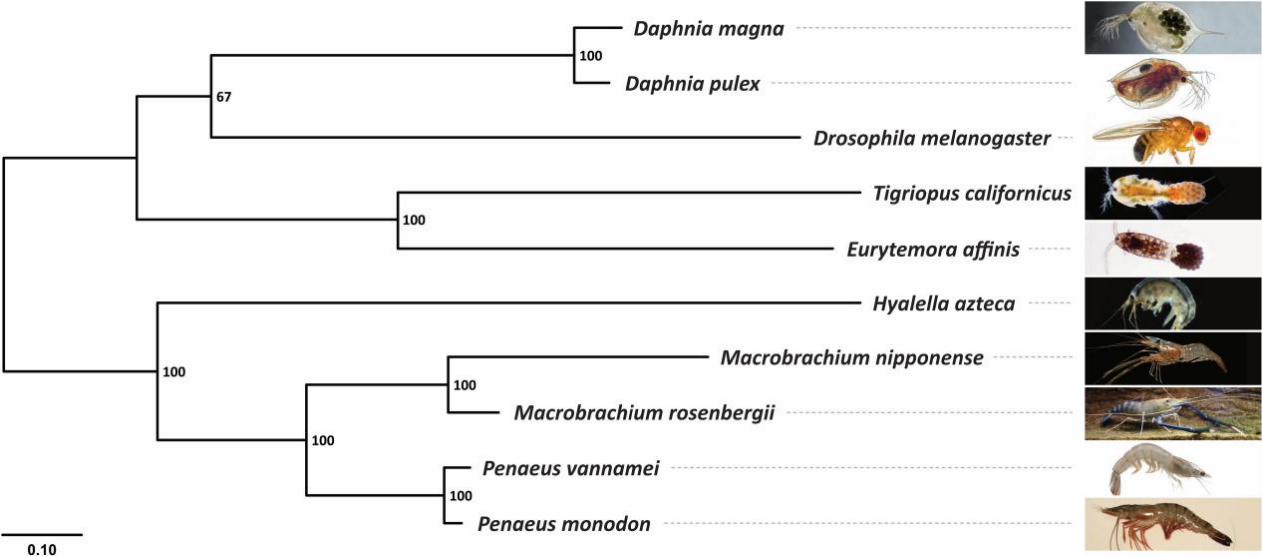
A maximum-likelihood tree of *M. rosenbergii* and related species constructed based on single-copy orthologous protein sequences. The numbers at each node represent the bootstrap values. The scale bar shows the number of substitutions per site.

To determine the phylogenetic relationship between *M. rosenbergii* and related species, we analyzed sequence information from single-copy orthologous genes from 10 Arthropod species: *Daphnia magna*, *Daphnia pulex*, *Drosophila melanogaster*, *Tigriopus californicus*, *Eurytemora affinis*, *H. azteca*, *M. nipponense, M. rosenbergii, L. vannamei*, and *P. monodon*. A maximum-likelihood phylogenetic tree obtained from clustering 251,370 proteins into 27,192 orthologous groups (251,370 input proteins from 10 species) suggested that *M. rosenbergii* had a close relationship with *M. nipponense* as well as 2 penaeid shrimps, *L. vannamei* and *P. monodon* (Fig. 3).

### Identification of alternative splicing variants

**Fig. 4.**
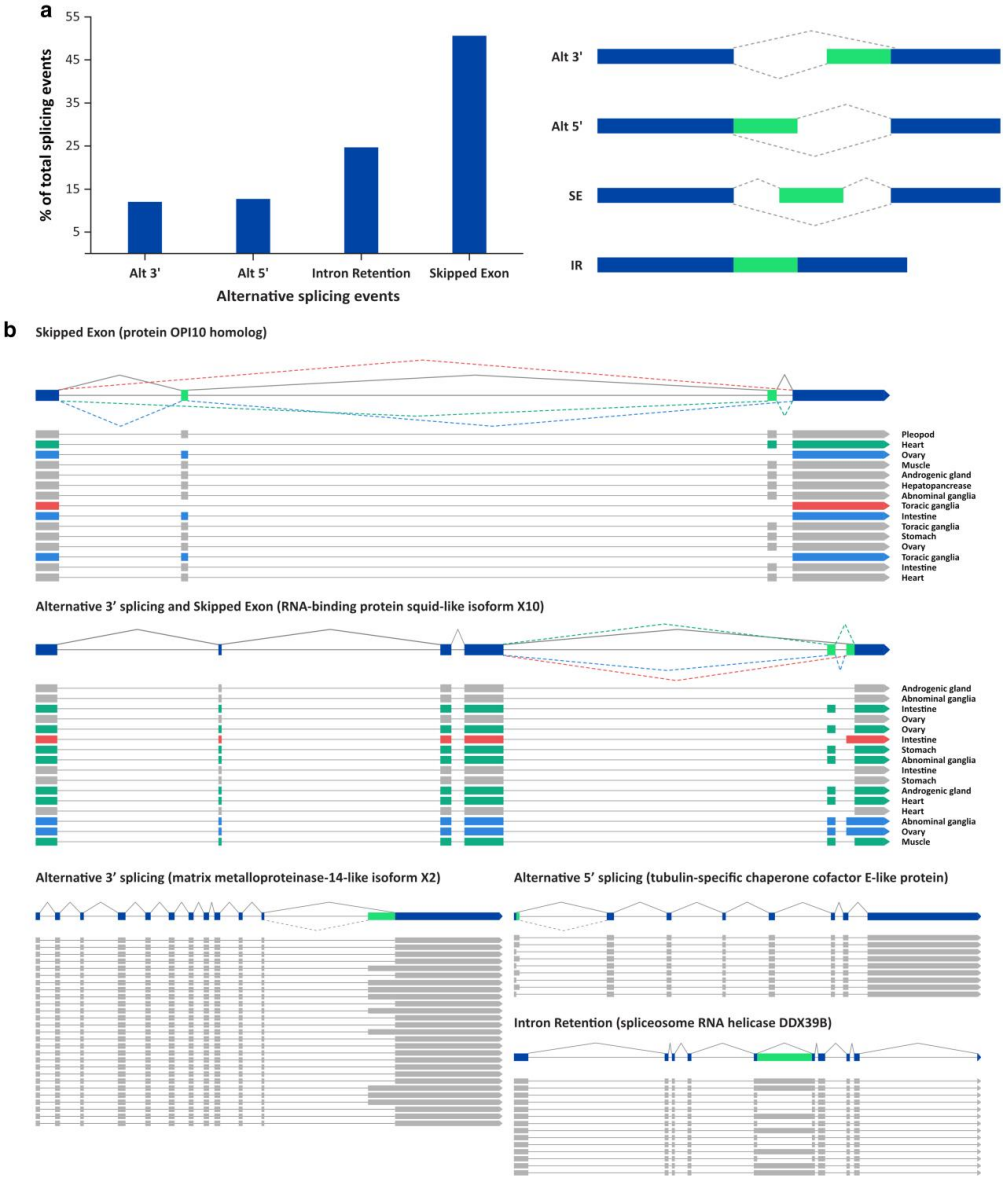
Alternative splicing in *M. rosenbergii*. a) The distribution of alternative splicing events. Alt 3′, alternative 3′ acceptor-site selection; Alt 5′, alternative 5′ donor-site selection; IR, intron retention; SE, skipped exon. b) Examples of transcripts with alternative splicing. Gene models displaying possible splicing events are shown at the top, and aligned Iso-seq reads are shown at the bottom. Protein OPI10 homolog and RNA-binding protein squid-like isoform X10 serve as examples of genes that undergo multiple alternative splicing events, with the tissue source of each Iso-seq read indicated. Various isoforms are distinguished by different colors.

The availability of both the chromosome-scale assembly and the full-length transcriptome assembly allowed us to thoroughly investigate the alternative splicing events in *M. rosenbergii*. We utilized the TAPIS pipeline (Abdel-Ghany *et al*. 2016) along with the SpliceGrapher program (Rogers *et al*. 2012) to thoroughly identify transcript variants exhibiting the following alternative splicing events: alternative 5′ donor-site selection, alternative 3′ acceptor-site selection, exon skipping, and intron retention. Our analysis revealed a total of 2,041 alternative splicing events in *M. rosenbergii* (Fig. 4a). Of these events, exon skipping was the most frequent, accounting for more than half (50.6%) of the total splicing events. The alternative 5′ donor-site selection and alternative 3′ acceptor-site selection appeared to be the least prevalent modes of alternative splicing, representing 12.7 and 12.0% of the total events identified, respectively. Finally, the occurrence of intron retention was observed in 24.6% of all alternative splicing events identified. Within individual genes, we observed different patterns of alternative splicing events in a combinatorial manner. Figure 4b depicts examples of these transcript isoforms containing multiple types of alternative splicing events. It is interesting to note that a specific isoform could be detected in multiple tissues. Furthermore, within a single tissue type, transcripts could undergo alternatively splicing, resulting in multiple isoforms.

## Discussion

*Macrobrachium rosenbergii* is an important aquaculture species widely cultivated in Southeast Asia. In our study, we successfully sequenced and assembled the reference genome of *M. rosenbergii*, utilizing the PacBio HiFi sequencing together with the Hi-C technique. We achieved a chromosome-scale assembly of the giant freshwater prawn genome encompassing a total of 3.73 Gb. Our assembly contains 59 pseudochromosomes corresponding to *M. rosenbergii*'s haploid chromosome number. The completeness of the gene space evaluated using BUSCO software demonstrated that our assembly contained 90.9% of the highly conserved orthologs in the Arthropod lineage. Compared with a related *Macrobrachium* species, the number of predicted genes in the *M. rosenbergii* (34,203) genome was lower than that in *M. nipponense* (44,086). The assembly revealed no evidence supporting a recent whole-genome duplication event in *M. rosenbergii*, contrary to a previously reported occurrence in *M. nipponense* (Jin *et al*. 2021). One remarkable feature of the *M. rosenbergii* genome was the unusually high occurrence of repetitive sequences, which occupied roughly 57% of the assembly (2.05 Gb). We observed a similar expansion of the repeatome in the oriental river prawn (*M. nipponese*; 2.11 Gb). The substantial accumulation of transposable elements in the *Macrobrachium* species likely contributes to the expansion of their genomes compared with the related penaeid shrimps (Fig. 3). In *M. rosenbergii*, the main category of repetitive elements that occupied almost half of its repeatome was the simple sequence repeats (42%; 904 Mb), comparable with the percentages observed in *L. vannamei* (51%; 453 Mb) and *P. monodon* (38%; 572 Mb).

The availability of our full-length transcript data from multiple tissues enabled us to investigate different forms of alternative splicing events, which have been known to regulate cell differentiation, development, and stress adaptation (Singh and Ahi 2022). Similar to the observations in Pacific white shrimp (Zhang *et al*. 2023), Pacific oyster (Huang *et al*. 2016), and fruit fly (Jakšić and Schlötterer 2016), exon skipping was the predominant alternative splicing event in the giant freshwater prawn. However, while intron retention represented the least frequent events in Pacific white shrimp, Pacific oyster, and fruit fly, alternative 3′ acceptor-site and alternative 5′ donor-site selections were the least prevalent modes of alternative splicing in *M. rosenbergii.* We strongly believe that the availability of a chromosome-scale reference genome for *M. rosenbergii* will play a pivotal role in advancing our understanding of the giant freshwater prawn biology and greatly facilitating its molecular breeding programs that ultimately lead to the development of *M. rosenbergii* with commercially desirable characteristics.

## Supplementary Material

jkae146_Supplementary_Data

## Data Availability

*Macrobrachium rosenbergii* genome assembly data have been submitted to the DDBJ/EMBL/GenBank databases under BioProject PRJNA875678 and PRJNA848969 and the following accession numbers: JANJGK000000000 (genome assembly; uploaded to GSA FigShare), SRR24764254 (PacBio subread data), SRR21415486 (PacBio CCS data), and SRR21415485 (Hi-C sequence data). The Iso-seq data have been submitted under BioProject PRJNA817196 and the following accession numbers: SRR18347406 (abdominal ganglia), SRR18347407 (thoracic ganglia), SRR18347408 (intestine), SRR18347409 (stomach), SRR18347410 (hepatopancreas), SRR18347411 (androgenic gland), SRR18347412 (testis), SRR18347413 (heart), SRR18347414 (ovary), SRR18347415 (muscle), SRR18347416 (pleopod), SRR18347417 (eyestalk), SRR18347418 (gill), and SRR18347419 (HC). Scripts for the computational steps are available at: https://github.com/NOC2024TH/Macrobrachium_Rosenbergii_genome_assembly. Supplemental material available at G3 online.

## References

[jkae146-B1] Abdel-Ghany SE , Hamilton M, Jacobi JL, Ngam P, Devitt N, Schilkey F, Ben-Hur A, Reddy AS. 2016. A survey of the sorghum transcriptome using single-molecule long reads. Nat Commun. 7(1):11706. doi:10.1038/ncomms11706. 27339290 PMC4931028

[jkae146-B2] Angthong P , Uengwetwanit T, Pootakham W, Sittikankaew K, Sonthirod C, Sangsrakru D, Yoocha T, Nookaew I, Wongsurawat T, Jenjaroenpun P, et al 2020. Optimization of high molecular weight DNA extraction methods in shrimp for a long-read sequencing platform. PeerJ. 8:e10340. doi:10.7717/peerj.10340. 33240651 PMC7668203

[jkae146-B3] Bao W , Kojima KK, Kohany O. 2015. Repbase update, a database of repetitive elements in eukaryotic genomes. Mob DNA. 6(1):11. doi:10.1186/s13100-015-0041-9. 26045719 PMC4455052

[jkae146-B4] Blum M , Chang HY, Chuguransky S, Grego T, Kandasaamy S, Mitchell A, Nuka G, Paysan-Lafosse T, Qureshi M, Raj S, et al 2021. The InterPro protein families and domains database: 20 years on. Nucleic Acids Res. 49(D1):D344–D354. doi:10.1093/nar/gkaa977. 33156333 PMC7778928

[jkae146-B5] Cao J , Wu L, Jin M, Li T, Hui K, Ren Q. 2017. Transcriptome profiling of the *Macrobrachium rosenbergii* lymphoid organ under the white spot syndrome virus challenge. Fish Shellfish Immunol. 67:27–39. doi:10.1016/j.fsi.2017.05.059. 28554835

[jkae146-B6] Carneiro CM , Noble JD, Pietras A, Moler P, Austin JD. 2021. Iso-seq analysis and functional annotation of the Santa Fe cave crayfish (*Procambarus erythrops*) transcriptome. Mar Genomics. 58:100842. doi:10.1016/j.margen.2021.100842. 34217485

[jkae146-B7] Cheng H , Concepcion GT, Feng X, Zhang H, Li H. 2021. Haplotype-resolved de novo assembly using phased assembly graphs with hifiasm. Nat Methods. 18(2):170–175. doi:10.1038/s41592-020-01056-5. 33526886 PMC7961889

[jkae146-B8] Ding Z , Jin M, Ren Q. 2018. Transcriptome analysis of *Macrobrachium rosenbergii* intestines under the white spot syndrome virus and poly (I:C) challenges. PLoS One. 13(9):e0204626. doi:10.1371/journal.pone.0204626. 30265693 PMC6161888

[jkae146-B9] Edgar RC . 2004. MUSCLE: multiple sequence alignment with high accuracy and high throughput. Nucleic Acids Res. 32(5):1792–1797. doi:10.1093/nar/gkh340. 15034147 PMC390337

[jkae146-B10] Emms DM , Kelly S. 2019. OrthoFinder: phylogenetic orthology inference for comparative genomics. Genome Biol. 20(1):238. doi:10.1186/s13059-019-1832-y. 31727128 PMC6857279

[jkae146-B11] FAOSTAT . [cited 2014 Dec 9]. Available from https://www.fao.org/faostat/en/#home.

[jkae146-B12] Flynn JM , Hubley R, Goubert C, Rosen J, Clark AG, Feschotte C, Smit AF. 2020. RepeatModeler2 for automated genomic discovery of transposable element families. Proc Natl Acad Sci U S A. 117(17):9451–9457. doi:10.1073/pnas.1921046117. 32300014 PMC7196820

[jkae146-B13] Fu L , Niu B, Zhu Z, Wu S, Li W. 2012. CD-HIT: accelerated for clustering the next-generation sequencing data. Bioinformatics. 28(23):3150–3152. doi:10.1093/bioinformatics/bts565. 23060610 PMC3516142

[jkae146-B14] Gao X , Jiang Z, Zhang S, Chen Q, Tong S, Liu X, Jiang Q, Yang H, Wei W, Zhang X. 2020. Transcriptome analysis and immune-related genes expression reveals the immune responses of *Macrobrachium rosenbergii* infected by *Enterobacter cloacae*. Fish Shellfish Immunol. 101:66–77. doi:10.1016/j.fsi.2020.03.042. 32213315

[jkae146-B15] Guan D , McCarthy SA, Wood J, Howe K, Wang Y, Durbin R. 2020. Identifying and removing haplotypic duplication in primary genome assemblies. Bioinformatics. 36(9):2896–2898. doi:10.1093/bioinformatics/btaa025. 31971576 PMC7203741

[jkae146-B16] Guo X-f , Zhou Y-l, Liu M, Wang Z-w, Gui J-f. 2022. Integrated application of Iso-seq and RNA-seq provides insights into unsynchronized growth in red swamp crayfish (*Procambarus clarkii*). Aquacult Rep. 22:101008. doi:10.1016/j.aqrep.2022.101008.

[jkae146-B17] Haas BJ , Salzberg SL, Zhu W, Pertea M, Allen JE, Orvis J, White O, Buell CR, Wortman JR. 2008. Automated eukaryotic gene structure annotation using EVidenceModeler and the program to assemble spliced alignments. Genome Biol. 9(1):R7. doi:10.1186/gb-2008-9-1-r7. 18190707 PMC2395244

[jkae146-B19] Huang B , Zhang L, Tang X, Zhang G, Li L. 2016. Genome-wide analysis of alternative splicing provides insights into stress adaptation of the pacific oyster. Mar Biotechnol (NY). 18(5):598–609. doi:10.1007/s10126-016-9720-x. 27771778

[jkae146-B18] Huang X , Adams MD, Zhou H, Kerlavage AR. 1997. A tool for analyzing and annotating genomic sequences. Genomics. 46(1):37–45. doi:10.1006/geno.1997.4984. 9403056

[jkae146-B20] Ibrahim S , Zhong Z, Lan X, Luo J, Tang Q, Xia Z, Yi S, Yang G. 2023. Morphological diversity of different male morphotypes of giant freshwater prawn *Macrobrachium rosenbergii* (De Man, 1879). Aquacult J. 3(2):133–148. doi:10.3390/aquacj3020012.

[jkae146-B21] Jakšić AM , Schlötterer C. 2016. The interplay of temperature and genotype on patterns of alternative splicing in *Drosophila melanogaster*. Genetics. 204(1):315–325. doi:10.1534/genetics.116.192310. 27440867 PMC5012396

[jkae146-B22] Jiang Q , Qian L, Gu S, Guo X, Zhang X, Sun L. 2020. Investigation of growth retardation in *Macrobrachium rosenbergii* based on genetic/epigenetic variation and molt performance. Comp Biochem Physiol Part D Genomics Proteomics. 35:100683. doi:10.1016/j.cbd.2020.100683. 32279060

[jkae146-B23] Jin S , Bian C, Jiang S, Han K, Xiong Y, Zhang W, Shi C, Qiao H, Gao Z, Li R, et al 2021. A chromosome-level genome assembly of the oriental river prawn, *Macrobrachium nipponense*. GigaScience. 10(1):giaa160. doi:10.1093/gigascience/giaa160. 33459341 PMC7812440

[jkae146-B24] Kanehisa M , Sato Y. 2020. KEGG mapper for inferring cellular functions from protein sequences. Protein Sci. 29(1):28–35. doi:10.1002/pro.3711. 31423653 PMC6933857

[jkae146-B25] Kriventseva EV , Tegenfeldt F, Petty TJ, Waterhouse RM, Simão FA, Pozdnyakov IA, Ioannidis P, Zdobnov EM. 2015. OrthoDB v8: update of the hierarchical catalog of orthologs and the underlying free software. Nucleic Acids Res. 43(D1):D250–D256. doi:10.1093/nar/gku1220. 25428351 PMC4383991

[jkae146-B26] Laetsch DR , Blaxter ML. 2017. BlobTools: interrogation of genome assemblies. F1000Res. 6:1287. doi:10.12688/f1000research.12232.1.

[jkae146-B27] Levy T , Rosen O, Manor R, Dotan S, Azulay D, Abramov A, Sklarz MY, Chalifa-Caspi V, Baruch K, Shechter A, et al 2019. Production of WW males lacking the masculine Z chromosome and mining the *Macrobrachium rosenbergii* genome for sex-chromosomes. Sci Rep. 9(1):12408. doi:10.1038/s41598-019-47509-6. 31455815 PMC6712010

[jkae146-B28] Li H . 2018. Minimap2: pairwise alignment for nucleotide sequences. Bioinformatics. 34(18):3094–3100. doi:10.1093/bioinformatics/bty191. 29750242 PMC6137996

[jkae146-B29] Li H , Durbin R. 2010. Fast and accurate long-read alignment with Burrows–Wheeler transform. Bioinformatics. 26(5):589–595. doi:10.1093/bioinformatics/btp698. 20080505 PMC2828108

[jkae146-B30] Liu Y , Hui M, Cui Z, Luo D, Song C, Li Y, Liu L. 2015. Comparative transcriptome analysis reveals sex-biased gene expression in Juvenile Chinese Mitten Crab Eriocheir sinensis. PLoS One. 10(7):e0133068. doi:10.1371/journal.pone.0133068. 26193085 PMC4507985

[jkae146-B31] Ma W , Ay F, Lee C, Gulsoy G, Deng X, Cook S, Hesson J, Cavanaugh C, Ware CB, Krumm A, et al 2018. Using DNase Hi-C techniques to map global and local three-dimensional genome architecture at high resolution. Methods. 142:59–73. doi:10.1016/j.ymeth.2018.01.014. 29382556 PMC5993575

[jkae146-B32] Marques HLA , New MB, Boock MV, Barros HP, Mallasen M, Valenti WC. 2016. Integrated freshwater prawn farming: state-of-the-art and future potential. Rev Fish Sci Aquacult. 24(3):264–293. doi:10.1080/23308249.2016.1169245.

[jkae146-B33] Moriya Y , Itoh M, Okuda S, Yoshizawa AC, Kanehisa M. 2007. KAAS: an automatic genome annotation and pathway reconstruction server. Nucleic Acids Res. 35(Web Server):W182–W185. doi:10.1093/nar/gkm321. 17526522 PMC1933193

[jkae146-B34] Nawrocki EP , Eddy SR. 2013. Infernal 1.1: 100-fold faster RNA homology searches. Bioinformatics. 29(22):2933–2935. doi:10.1093/bioinformatics/btt509. 24008419 PMC3810854

[jkae146-B35] Ou J , Chen H, Liu Q, Bian Y, Luan X, Jiang Q, Ji H, Wang Z, Lv L, Dong X, et al 2021. Integrated transcriptome analysis of immune-related mRNAs and microRNAs in *Macrobrachium rosenbergii* infected with *Spiroplasma eriocheiris*. Fish Shellfish Immunol. 119:651–669. doi:10.1016/j.fsi.2021.11.002. 34742900

[jkae146-B36] Pootakham W , Uengwetwanit T, Sonthirod C, Sittikankaew K, Karoonuthaisiri N. 2020. A novel full-length transcriptome resource for black tiger shrimp (*Penaeus monodon*) developed using isoform sequencing (Iso-Seq). Front Mar Sci. 7:172. doi:10.3389/fmars.2020.00172.

[jkae146-B37] Putnam NH , O'Connell BL, Stites JC, Rice BJ, Blanchette M, Calef R, Troll CJ, Fields A, Hartley PD, Sugnet CW, et al 2016. Chromosome-scale shotgun assembly using an in vitro method for long-range linkage. Genome Res. 26(3):342–350. doi:10.1101/gr.193474.115. 26848124 PMC4772016

[jkae146-B38] Ren J , Na R, Chen H, Lou B, Niu B. 2021. RNA sequencing and functional analysis of adult gonadal tissue to identify candidate key genes in *Macrobrachium rosenbergii* sex development. Aquacult Int. 29(6):2805–2821. doi:10.1007/s10499-021-00780-9.

[jkae146-B39] Rogers MF , Thomas J, Reddy ASN, Ben-Hur A. 2012. SpliceGrapher: detecting patterns of alternative splicing from RNA-Seq data in the context of gene models and EST data. Genome Biol. 13(1):R4. doi:10.1186/gb-2012-13-1-r4. 22293517 PMC3334585

[jkae146-B40] Simão FA , Waterhouse RM, Ioannidis P, Kriventseva EV, Zdobnov EM. 2015. BUSCO: assessing genome assembly and annotation completeness with single-copy orthologs. Bioinformatics. 31(19):3210–3212. doi:10.1093/bioinformatics/btv351. 26059717

[jkae146-B41] Singh P , Ahi EP. 2022. The importance of alternative splicing in adaptive evolution. Mol Ecol. 31(7):1928–1938. doi:10.1111/mec.16377. 35094439

[jkae146-B42] Stanke M , Steinkamp R, Waack S, Morgenstern B. 2004. AUGUSTUS: a web server for gene finding in eukaryotes. Nucleic Acids Res. 32(Web Server):W309–W312. doi:10.1093/nar/gkh379. 15215400 PMC441517

[jkae146-B43] Tamura K , Peterson D, Peterson N, Stecher G, Nei M, Kumar S. 2011. MEGA5: molecular evolutionary genetics analysis using maximum likelihood, evolutionary distance, and maximum parsimony methods. Mol Biol Evol. 28(10):2731–2739. doi:10.1093/molbev/msr121. 21546353 PMC3203626

[jkae146-B44] Tang Q , Xia Z, Cai M, Du H, Yang J, Xu Y, Zhang H, Li J, Wu Y, Xie J, et al 2020. Identification of differentially expressed genes and signalling pathways to elucidate molecular mechanisms underlying growth differences among the male morphotypes of *Macrobrachium rosenbergii*. Aquac Res. 51(12):5040–5049. doi:10.1111/are.14842.

[jkae146-B45] Tempel S . 2012. Using and understanding RepeatMasker. Methods Mol Biol. 859:29–51. doi:10.1007/978-1-61779-603-6_2. 22367864

[jkae146-B47] Wu TD , Watanabe CK. 2005. GMAP: a genomic mapping and alignment program for mRNA and EST sequences. Bioinformatics. 21(9):1859–1875. doi:10.1093/bioinformatics/bti310. 15728110

[jkae146-B46] Wu Y , Li Y, Wang R, Zhao Y, Liu H, Wang JJ. 2021. Characterization of a novel food grade emulsion stabilized by the by-product proteins extracted from the head of giant freshwater prawn (*Macrobrachium rosenbergii*). Front Nutr. 8:676500. doi:10.3389/fnut.2021.676500. 34249988 PMC8266994

[jkae146-B48] Yang G , Frinsko M, Chen X, Wang J, Hu G, Gao Q. 2012. Current status of the giant freshwater prawn (*Macrobrachium rosenbergii*) industry in China, with special reference to live transportation. Aquac Res. 43(7):1049–1055. doi:10.1111/j.1365-2109.2011.03009.x.

[jkae146-B49] Ying N , Wang Y, Song X, Qin B, Wu Y, Yang L, Fang W. 2022. Transcriptome analysis of *Macrobrachium rosenbergii*: identification of precocious puberty and slow-growing information. J Invertebr Pathol. 190:107752. doi:10.1016/j.jip.2022.107752. 35367462

[jkae146-B50] Zhang X , Zhang X, Yuan J, Li F. 2023. The responses of alternative splicing during heat stress in the pacific white shrimp *Litopenaeus vannamei*. Genes (Basel). 14(7):1473. doi:10.3390/genes14071473. 37510377 PMC10379218

