# Supplementary material for: A chromosome-level reference genome assembly and a full-length transcriptome assembly of the giant freshwater prawn (Macrobrachium rosenbergii)
Source: G3 (Bethesda). 2024 Jul 8;14(9):jkae146. doi: 10.1093/g3journal/jkae146 (PMC11373640; doi:10.1093/g3journal/jkae146)
Supplement: jkae146_Supplementary_Data [file jkae146_supplementary_data.zip › Supplemental_Figures_G3-2024-405190.pptx]

## Slide 1
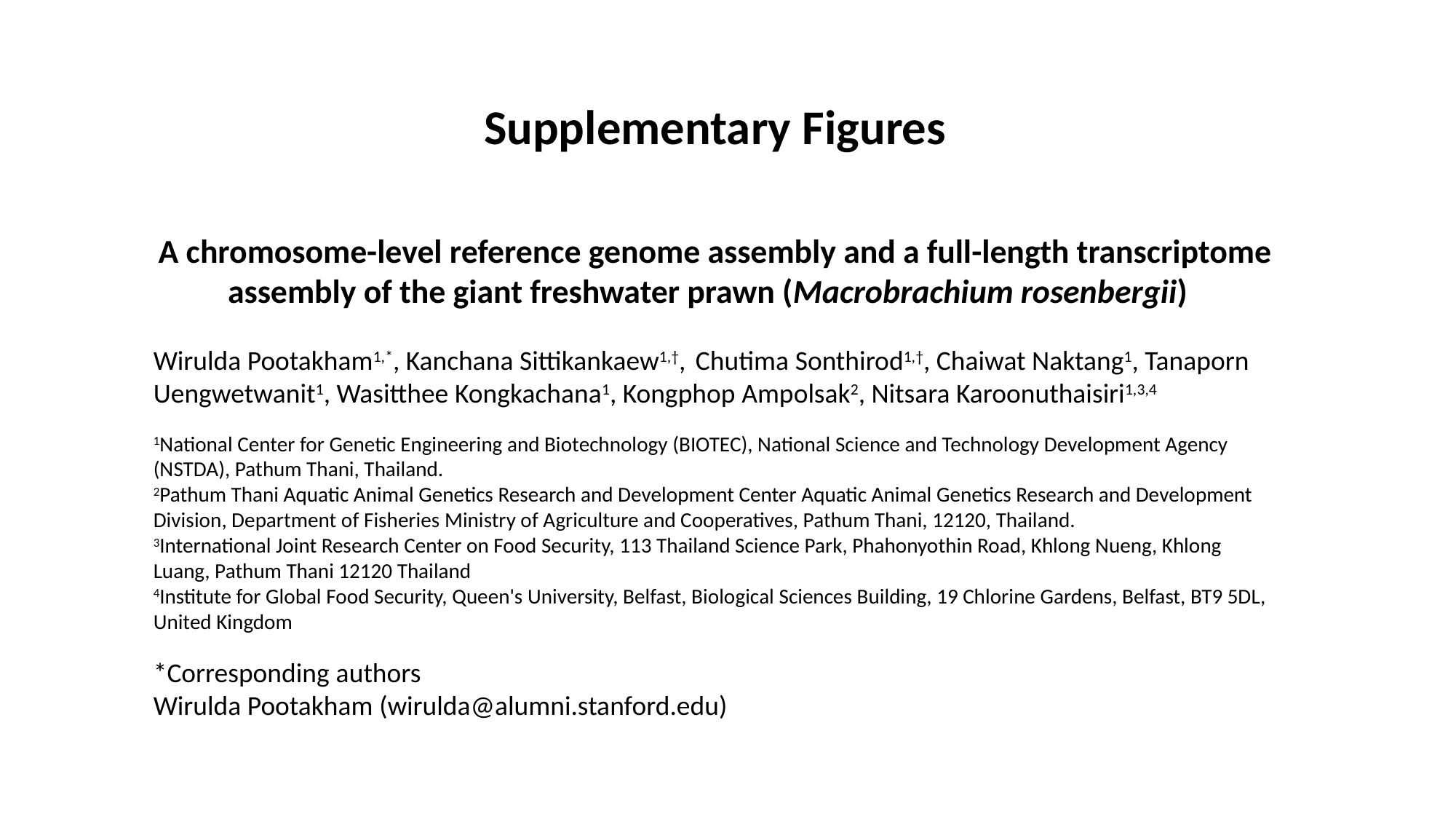

Supplementary Figures
A chromosome-level reference genome assembly and a full-length transcriptome assembly of the giant freshwater prawn (Macrobrachium rosenbergii)
Wirulda Pootakham1,*, Kanchana Sittikankaew1,†, Chutima Sonthirod1,†, Chaiwat Naktang1, Tanaporn Uengwetwanit1, Wasitthee Kongkachana1, Kongphop Ampolsak2, Nitsara Karoonuthaisiri1,3,4
1National Center for Genetic Engineering and Biotechnology (BIOTEC), National Science and Technology Development Agency (NSTDA), Pathum Thani, Thailand.
2Pathum Thani Aquatic Animal Genetics Research and Development Center Aquatic Animal Genetics Research and Development Division, Department of Fisheries Ministry of Agriculture and Cooperatives, Pathum Thani, 12120, Thailand.
3International Joint Research Center on Food Security, 113 Thailand Science Park, Phahonyothin Road, Khlong Nueng, Khlong Luang, Pathum Thani 12120 Thailand
4Institute for Global Food Security, Queen's University, Belfast, Biological Sciences Building, 19 Chlorine Gardens, Belfast, BT9 5DL, United Kingdom
*Corresponding authors
Wirulda Pootakham (wirulda@alumni.stanford.edu)

## Slide 2
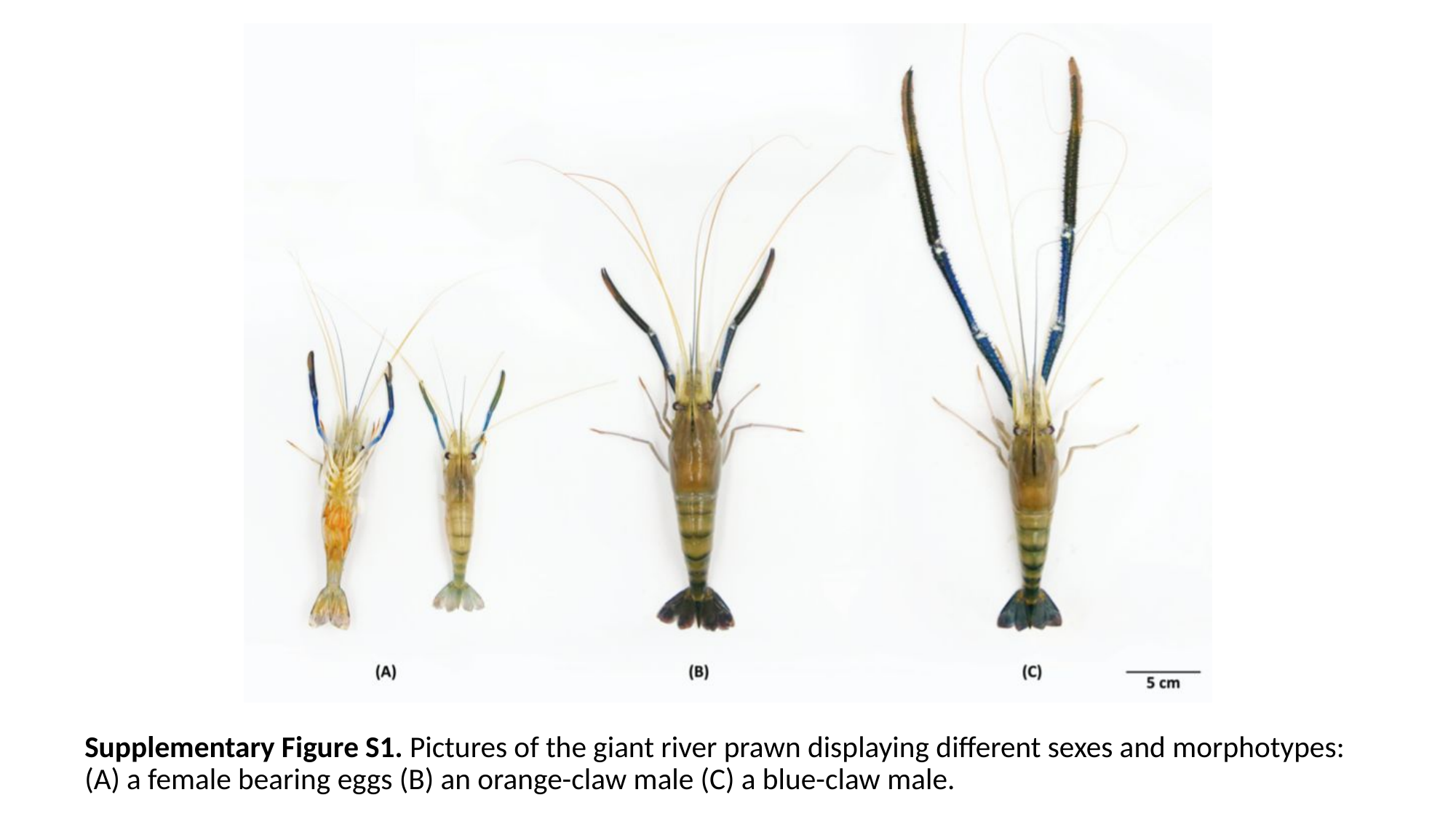

Supplementary Figure S1. Pictures of the giant river prawn displaying different sexes and morphotypes: (A) a female bearing eggs (B) an orange-claw male (C) a blue-claw male.

## Slide 3
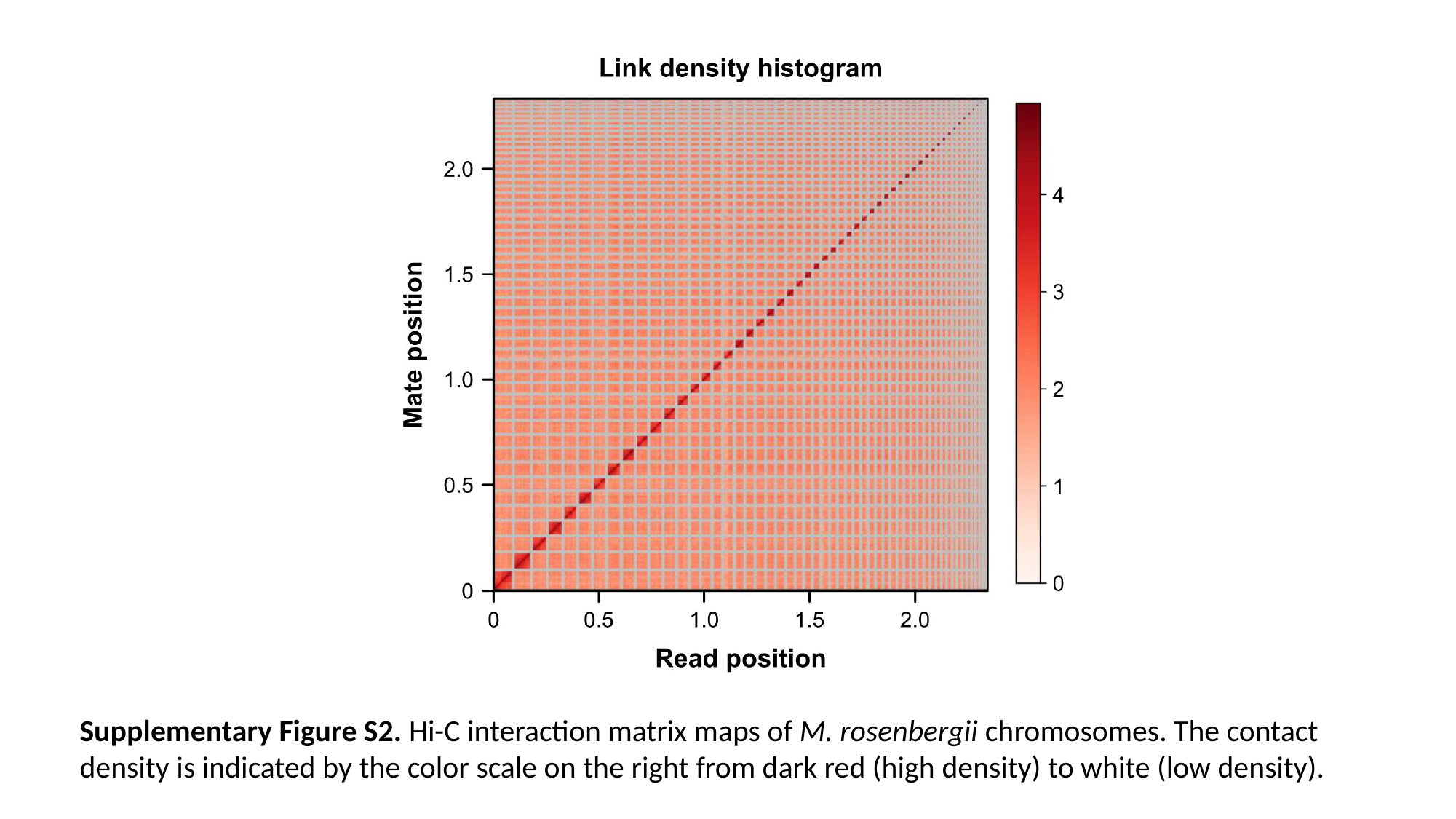

Supplementary Figure S2. Hi-C interaction matrix maps of M. rosenbergii chromosomes. The contact density is indicated by the color scale on the right from dark red (high density) to white (low density).

## Slide 4
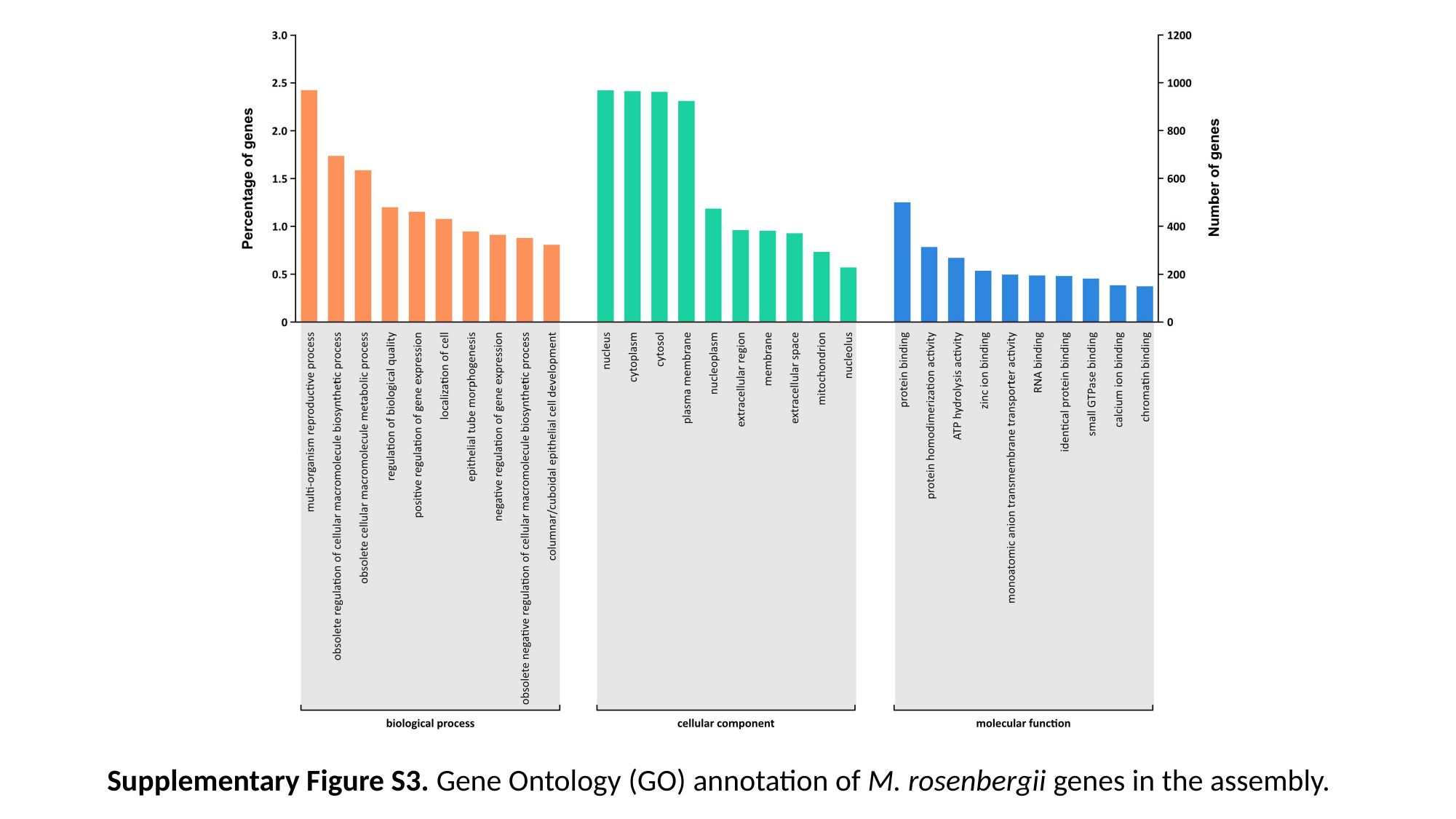

Supplementary Figure S3. Gene Ontology (GO) annotation of M. rosenbergii genes in the assembly.
